# Associative memory in alcohol-related contexts: An fMRI study with young binge drinkers

**DOI:** 10.1177/02698811241282624

**Published:** 2024-10-07

**Authors:** Rui Pedro Serafim Rodrigues, Sónia Silva Sousa, Eduardo López-Caneda, Natália Almeida-Antunes, Alberto Jacobo González‑Villar, Adriana Sampaio, Alberto Crego

**Affiliations:** University of Minho, Braga, Portugal

**Keywords:** Binge drinking, associative memory, contextual cues, attentional bias, neuroimaging

## Abstract

**Background:**

Alcohol-related cues are known to influence craving levels, a hallmark of alcohol misuse. Binge drinking (BD), a pattern of heavy alcohol use, has been associated with cognitive and neurofunctional alterations, including alcohol attentional bias, memory impairments, as well as disrupted activity in prefrontal- and reward-related regions. However, literature is yet to explore how memories associated with alcohol-related cues are processed by BDs, and how the recall of this information may influence their reward processing.

**Aims:**

The present functional magnetic resonance imaging (fMRI) study aimed to investigate the neurofunctional signatures of BD during an associative memory task.

**Method:**

In all, 36 university students, 20 BDs and 16 alcohol abstainers, were asked to memorize neutral objects paired with either alcohol or non-alcohol-related contexts. Subsequently, neutral stimuli were presented, and participants were asked to classify them as being previously paired with alcohol- or non-alcohol-related contexts.

**Results:**

While behavioral performance was similar in both groups, during the recall of alcohol-related cues, BDs showed increased brain activation in two clusters including the thalamus, globus pallidus and dorsal striatum, and cerebellum and occipital fusiform gyrus, respectively.

**Conclusion:**

These findings suggest that BDs display augmented brain activity in areas responsible for mental imagery and reward processing when trying to recall alcohol-related cues, which might ultimately contribute to alcohol craving, even without being directly exposed to an alcohol-related context. These results highlight the importance of considering how alcohol-related contexts may influence alcohol-seeking behavior and, consequently, the maintenance or increase in alcohol use.

## Introduction

Alcohol use is the seventh leading risk factor for premature death, with hazardous consumption patterns contributing to over 5% of all global deaths (Griswold et al., 2018; World Health Organization (WHO), 2019). Particularly, among young people, alcohol use plays a significant social role, being an integral part of academic traditions and parties (Dormal et al., 2019; Kuntsche et al., 2005; Patrick et al., 2016). This social aspect of alcohol consumption is particularly concerning, as alcohol misuse is linked to more than 30% of deaths among individuals aged 15–29 years in both America and Europe (WHO, 2011).

Binge drinking (BD) is a recurrent alcohol consumption pattern characterized by alternating episodes of excessive alcohol consumption and periods of abstinence or low consumption. During BD episodes, individuals reach a blood alcohol concentration of 0.08 g/dL or higher, typically achieved by consuming five or more drinks for cisgender men and four or more drinks for cisgender women within a 2-h interval (National Institute on Alcohol Abuse and Alcoholism (NIAAA), 2004, 2022). The periods of abstinence or low consumption can last from several days to several weeks, leading to intoxication/abstinence cycles (Maurage et al., 2020). In addition, it is common to establish as a criterion for this pattern that BD episodes should occur at least once or twice per month during the last 12 months (Courtney & Polich, 2009; Maurage et al., 2020; NIAAA, 2022; Parada et al., 2011). This consumption pattern is a problematic form of alcohol misuse highly prevalent among young people, and notably widespread among European and American college students, with more than one-third engaging in recurrent BD episodes at least once a month (Kraus and Nociar, 2016; NIAAA, 2020). This highlights the concerning prevalence of BD in this population and underscores the significant impact of the college environment on alcohol consumption (Cadigan et al., 2015).

The high prevalence of BD during adolescence and youth is particularly worrying due to the critical neuromaturational processes occurring at these developmental stages (Jones et al., 2018; Sousa et al., 2018). Namely, structural and functional brain changes, such as synaptic pruning and myelination, take place in regions of late neuromaturation like the prefrontal cortex, an area crucial for the development of higher-level cognitive functions, including working memory, inhibitory control, and decision-making (Fuhrmann et al., 2015; Gogtay et al., 2004; Luna et al., 2010). These changes render the brain highly vulnerable to the neurotoxic effects of alcohol (Crews et al., 2016). Specifically, alcohol interferes with these processes by disrupting the formation of new neural connections and damaging existing neural pathways, leading to long-term cognitive deficits and impaired brain function (Jacobus and Tapert, 2013). This disruption can result not only in reduced cognitive abilities but also poor academic performance and an increased risk of developing psychiatric disorders (Castellanos-Ryan and Conrod, 2020).

BD has often been associated with enhanced impulsivity and reduced inhibitory control (Crews & Boettiger, 2009; Cservenka & Brumback, 2017; Dalley & Robbins, 2017; López-Caneda et al., 2014a). These behavioral alterations have been linked to abnormalities in the frontostriatal system (see Pérez-García et al., 2022, for a review). In particular, studies have found increased volume in the dorsolateral prefrontal cortex, nucleus accumbens, dorsal cingulate, and precuneus among individuals who engage in BD, suggesting that the structural alterations in these areas may underlie the impaired cognitive functions commonly observed in this population (Banca et al., 2016; Doallo et al., 2014; Sousa et al., 2017, 2020). In addition, binge drinkers (BDs) frequently exhibit brain hyperactivation when compared to their control peers (Cservenka & Brumback, 2017; Schacht et al., 2013; Sousa et al., 2019). Notably, increased prefrontal activity during behavioral or cognitive inhibition tasks has been interpreted as a neurocompensatory mechanism, that is, the recruitment of additional neural resources that allow BDs to perform the task at the same level as controls (Almeida-Antunes et al., 2024; Ames et al., 2014; Correas et al., 2021; López-Caneda et al., 2012; Molnar et al., 2018; Suárez-Suárez et al., 2020; Wetherill et al., 2013). Similarly, studies exploring memory processes in BDs have revealed increased frontoparietal and hippocampal activity during the novel and/or correct encoding of visual or verbal items (Dager et al., 2014; Schweinsburg et al., 2010). This suggests that the less efficient neural processing and compensatory mechanisms observed in inhibitory control are also present during working memory tasks. Moreover, stronger memory for alcohol-related associations, that is, better recall for alcohol-related associations was found to be predictive of heavier drinking in patients with alcohol use disorder (AUD) (Goldfarb et al., 2020), underscoring the importance of associative memory in understanding alcohol consumption in real-life scenarios.

Associative memory is defined as the ability to link together two or more memories/items (e.g., images, words, sounds, places) that can later be retrieved through recognition or recall of one of them (Mayes et al., 2007). In the context of alcohol use, this involves stimuli associated with alcohol-related contexts, such as bars, bottles of alcoholic beverages, people drinking and cheering, or logos of popular alcohol brands. These stimuli are often encountered in social settings and can trigger memories and associations related to drinking behavior, and alcohol-related cues can elicit craving and influence drinking behavior, a phenomenon well documented in individuals with AUD (Seo et al., 2014). By contrast, neutral cues are stimuli that have no direct connection with alcohol consumption. These might include everyday objects, like books, pens, or images of neutral environments, such as a park or a classroom.

Although no study to date has explored brain activity linked to associative memory involving both neutral and alcohol-related cues in young BDs, evidence indicates that the neurofunctional correlates of associative memory are similar to those observed in craving, the formation of consumption habits, and alcohol cue-induced reactivity in heavy alcohol users (Courtney et al., 2016; Kim, 2011; Janak & Chaudhri, 2010; Park et al., 2007; Schacht et al., 2013; Scimeca & Badre, 2012). Specifically, encoding of pairs of images involves activation of a widely spread brain network, including the fusiform gyrus, the precuneus, the hippocampus, and the prefrontal and occipital cortices (Becker et al., 2015; de Chastelaine et al., 2016; Kim, 2011). Memory retrieval, on the other hand, activates regions such as the prefrontal cortex, striatum, and hippocampus (Schott et al., 2019; Scimeca & Badre, 2012). In addition, the amygdala, the precuneus, and sensory regions including the fusiform gyrus and the occipital cortex, recruited during encoding, are often reactivated during recall, particularly if the memorized information has emotional significance or was encoded through mental imagery (Danker & Anderson, 2010). Likewise, research has shown that heavy alcohol users display higher cue-reactivity to alcohol stimuli, exhibiting increased brain activity in the prefrontal (e.g., ventromedial and dorsolateral prefrontal cortex) and visual (e.g., fusiform gyrus and precuneus) regions as well as in several structures of the limbic system, including the striatum and the thalamus (Courtney et al., 2016; Fukushima et al., 2020; Huang et al., 2018; Park et al., 2007; Schacht et al., 2013). In a related vein, the mechanisms underlying cue-induced craving, commonly observed in addiction (Heinz et al., 2009; Witteman et al., 2015), have been closely related to associative memory (Janak & Chaudhri, 2010). Both processes entail the linking of two or more stimuli—whether internal or external—that can later be recalled, either intentionally or spontaneously, upon encountering one of them (Pershin & Di Ventra, 2010; Wrase et al., 2007).

Notwithstanding, research on the relation between associative memory and the processes underlying alcohol craving (e.g., attentional bias, reward processing, habit formation) and how these interact with alcohol use is still scarce. Aiming to fill this gap, the present study aimed to explore the brain mechanisms involved in the recall of alcohol-related contexts in young BDs. Hence, we conducted a whole-brain functional magnetic resonance imaging (fMRI) analysis and compared cue-induced brain activation between BDs and alcohol abstainers when recalling alcoholic or non-alcoholic contexts in response to associated neutral stimuli. According to previous research showing augmented brain activity in the reward and the fronto occipital networks during both the processing of alcohol-related cues and encoding/recovery of visual memories (Almeida-Antunes et al., 2022; Brumback et al., 2015; Courtney et al., 2015; Dager et al., 2014), we hypothesize that BDs will display increased activation of the reward and memory brain regions when recalling alcohol-related contexts.

## Method

### Participants

Participants aged between 18 and 23 years were recruited via an online survey and divided into two groups. Inclusion criteria for the BD group required participants to have consumed four or more drinks for women (five or more for men) in less than 2 h at least once per month for the past 10 months, ensuring a consistent pattern of BD. The second group served as the control group, consisting of alcohol abstainers without a drinking history; that is, they had never consumed alcohol and, thus, had never experienced a BD episode in their lives. Preselected participants completed a clinical interview including different questionnaires (see Table 1), which assessed their eligibility to participate in the study. Participants were excluded from either group based on the following criteria: a score of 20 or higher on the Alcohol Use Disorders Identification Test (AUDIT), indicating alcohol abuse or dependence; left-handedness, as indicated by a score of −40 or lower on the Edinburgh Handedness Inventory; a Global Severity Index score ⩾90, or a score ⩾90 on at least two symptomatic dimensions of the SCL-90-R; uncorrected sensory deficits (e.g., participants with poor eyesight who do not use glasses or contact lenses); a personal history of traumatic brain injury or neurological disorder; regular cannabis consumption (i.e., weekly use); a personal history of regular or occasional use of other drugs, including opiates, hallucinogens, cocaine, ecstasy, amphetamines, or medically prescribed psychoactive substances; alcohol abuse or dependence as per DSM-IV-R criteria; a family history of any neurological disorder or DSM-IV axis I disorder in first-degree relatives; a family history of substance abuse, including alcohol; and any contraindications for magnetic resonance imaging (e.g., metal implants, metallic objects, pregnancy, recent surgeries, or any type of medical condition that would prevent them from entering the scanner safely).

**Table 1.** Scales and questionnaires used in the clinical interview.

Portuguese version of the Alcohol Use Disorder Identification Test (AUDIT) (Babor et al., 2001; da Cunha, 2002)
Alcohol Use Questionnaire—items 10, 11, and 12 (Townshend and Duka, 2002)
Semi-structured assessment for the genetics of alcoholism, the individual assessment module, and the family history assessment module (Bucholz et al., 1994)
Portuguese version of the Symptom Checklist-90 Revised (SCL-90-R) (Derogatis and Unger, 2010; Laloni, 2001)
Portuguese version of Barratt Impulsiveness Scale 11 (BIS11) (Patton et al., 1995)
Edinburg handedness inventory (Oldfield, 1971)

Thus, the final sample included 36 college students: 20 BDs (50% female) and 16 alcohol-abstinent individuals (62.5% female). See Table 2 for complete demographic, drinking characteristics, and psychological traits.

**Table 2.** Demographic, drinking characteristics, and psychological traits of the BD and control groups (mean (SD)).

	Controls mean ± SD	Binge drinkers mean ± SD	*t* (34)
Demographic measures			
*N* (female)	16 (10)	20 (10)	
Caucasian ethnicity (%)	100	100	
Age	21.00 (1.71)	20.45 (1.60)	−0.99
Age of BD onset	—	17.45 ± 1.08	
Drinking characteristics			
AUDIT (total score)	0.62 ± 1.20	11.20 ± 3.25	13.43***
BD episodes per month	—	3.58 ± 1.87	8.54***
Percentage of alcohol intoxication per drinking event	—	43.25 ± 20.41	9.48***
Number of months with BD pattern	—	35.90 ± 14.03	11.44***
Quantity of alcohol consumed (gr/week)	—	151 ± 44.27	14.78***
Speed of drinking (gr/h during BD episodes)	—	34.50 ± 8.26	18.69***
Tobacco smokers (female)	—	7 (4)	
Occasional users of cannabis	—	2 (2)	
Psychological traits			
Impulsivity (BIS11)			
Total score	63.56 ± 6.05	64.80 ± 5.83	0.62
Attention	10.63 ± 2.16	10.65 ± 2.23	0.03
Cognitive instability	5.50 ± 1.51	6.10 ± 1.41	1.23
Motor	10.00 ± 2.03	11.80 ± 2.76	2.17*
Perseverance	7.31 ± 2.03	7.05 ± 1.05	–0.64
Self-control	17.38 ± 2.58	16.50 ± 2.46	–1.04
Cognitive complexity	12.75 ± 1.88	12.70 ± 1.95	–0.08
Psychopathological symptoms (SCL-90-R)			
Anxiety	0.78 ± 0.61	0.58 ± 0.58	1.01
Depression	1.01 ± 0.64	0.96 ± 0.60	0.22
Global severity index	0.62 ± 0.38	0.65 ± 0.41	−0.22

AUDIT: alcohol use disorders identification test; BIS11: Barratt Impulsiveness Scale 11; BD: binge drinking; SCL-90-R: Symptom checklist-90 revised; SD: standard deviation.

All *p*-values reported are for two-tailed independent samples *t*-tests. *p ⩽ 0.05. ***p ⩽ 0.001.

### Procedure

The research protocol followed the Code of Ethical Principles for Medical Research Involving Human Subjects of the World Medical Association outlined in the Declaration of Helsinki (World Medical Association, 2013), meeting the requirements for exemption and anonymity, and it was approved by the Ethics Committee for Research in Social and Human Sciences of the University of (details omitted for double-anonymized peer review).

### Task

**Figure 1.**
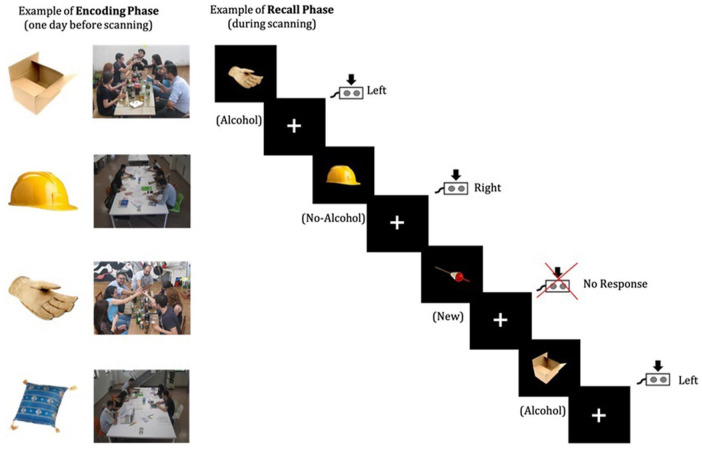
The overall depiction of the associative memory task for alcohol-related contexts. (a) Example of encoding phase: One day before scanning 90 pairs of images are memorized; (b) Example of recall phase: During scanning, participants had to classify each of the 135 neutral stimuli presented as being associated with either an alcohol or no-alcohol context or new stimuli.

An associative memory task was specially developed to examine memory retrieval mechanisms for alcohol-related contexts (see Figure 1), building upon the task previously developed to assess associative memory with neutral stimuli by Yonelinas et al. (2001). One day prior to the scanning, during the encoding phase, participants were asked to memorize 90 pairs of images. Each pair contained an image of an object and a picture of young people in one of two social contexts, that is, drinking (alcohol context) or studying (no-alcohol context). The social context pictures were created specifically for this task and object images were obtained from the POPORO database (Kovalenko et al., 2012), and classified (1–9) as neutral for emotional valence (*M* = 4.97) and arousal (*M* = 4.63). Participants were instructed to remember which of the two (alcohol/no-alcohol) contexts was associated with each object. Subsequently, participants were randomly presented the neutral stimuli and asked to indicate whether they were previously associated with an alcohol or a no-alcohol context. To guarantee that inside the scanner participants were indeed conducting associative memory processes, as well as to ensure homogeneity between individual learning effects, participants’ performance was tested the day before the scan, as well as immediately prior to entering the scanner. Participants who did not achieve a minimum of 90% of correct recalls on either occasion were asked to repeat the encoding phase until they reached the required threshold. Participants were then scanned while making recognition memory judgments. Specifically, they were presented with a list of 135 images, consisting of 90 memorized neutral objects and 45 new objects. Each stimulus was presented sequentially followed by a fixation cross. The trial sequence and presentation time of the stimulus and fixation cross were pseudorandomized and determined using the optseq2 toolbox (https://surfer.nmr.mgh.harvard.edu/optseq/). Participants were asked to observe each of the images presented and respond by pressing one of two buttons if they thought the item was previously presented—left for pictures associated with alcoholic contexts and right for pictures associated with no-alcohol contexts—and to not respond if they thought the item was new.

### Magnetic resonance image acquisition

Functional imaging was acquired with a Siemens Magneton TrioTrim 3T MRI scanner (Siemens Medical Solutions, Erlangen, Germany) equipped with a 32-channel receive-only coil. The blood oxygen level-dependent (BOLD) sensitive echo-planar imaging (EPI) was used with the following acquisition parameters: 39 interleaved axial slices; repetition time (TR) = 2000 ms; echo time (TE) = 29 ms; flip angle (FA) = 90º; slice thickness = 3 mm; voxel size = 3 mm × 3 mm × 3 mm; and field of view (FoV) = 222 mm. The acquisition was made in a block with a duration of 10.36 min, consisting of 311 volumes.

High-resolution 3D T1-weighted images were also acquired to coregister to the realigned mean functional image. To this end, a magnetization-prepared rapid acquisition gradient echo ascending interleaved sequence was applied using the following parameters: TR/TE = 2700/2.33 ms; inversion time = 1000 ms; delay time = 1600 ms; FA = 7°, 192 slices with 0.8 mm thickness; slab thickness = 153.6 mm; slice gap = 0 mm; in-plane resolution = 1 × 1 mm^2^; matrix size = 320 × 310; and 256 mm FoV.

### Image preprocessing and analysis

The fMRI data preprocessing was performed using Statistical Parametric Mapping (SPM12; https://www.fil.ion.ucl.ac.uk/spm/software/spm12/) implemented in Matlab (version 2021b, The Mathworks, Inc., Natick, MA, USA).

First, functional and anatomical images were reoriented to the anterior commissure. Subsequently, the intensity inhomogeneity was corrected using the field maps that were acquired during the MRI protocol. Then, functional images were corrected for slice timing and realigned and unwarped to correct movement artifacts (images with movements that were superior to voxel size were removed). The anatomical T1 images were coregistered to the realigned mean functional image and then images were transformed into standard MNI space using segmentation-based normalization parameters. Finally, the resulting functional images were spatially smoothed using an 8-mm FWHM Gaussian kernel, and low-frequency drifts were removed with a high-pass temporal filter (filter width of 128 s).

### Statistical analysis

#### Demographic data, drinking variables, and psychological traits comparisons

Differences between groups concerning demographics, drinking, and psychological traits (impulsivity and psychopathological symptomatology) and variables were compared using multiple two-sample *t*-tests. In addition, given the uneven number of female participants between the two groups, a Chi-square analysis was conducted to assess the potential impact of this distribution.

#### Behavioral analysis

During the memory test phase, items that had been previously presented in the encoding phase and correctly associated with the alcohol- or non-alcohol-related context were considered correct responses. Thus, the percentage of correct responses was computed according to the following formula: number of correctly recalled items/number of previously recalled items × 100.

Reaction times (RTs) and percentages of correct recalls for alcohol and no-alcohol images were statistically analyzed using two-way mixed-design ANOVA with one within-subjects factor (Condition: Alcohol and No-Alcohol) and one between-subjects factor (Group: BDs and Control) with IBM Statistical Package for Social Sciences (SPSS, v.28; IBM Corp., 2021, Armonk, NY, USA). Lastly, correct inhibitions for the New condition were analyzed using two-sample *t*-tests.

#### Neuroimaging analysis

The fMRI analyses were performed using the general linear model approach with SPM12 (Wellcome Centre for Human Neuroimaging, 2014, London, UK) software. The fMRI paradigm was analyzed by creating a set of regressors at the time of each decision-making, which were convolved with the hemodynamic response function. Three different regressors were created for each participant, one for each of the three conditions: “Alcohol” (trials with correct responses for images of neutral objects associated with alcohol contexts), “No-Alcohol” (trials with correct responses for images of neutral objects associated with study contexts), and “New” (trials with no responses for the new images of neutral objects). The six motion regressors estimated at the preprocessing stage and a regressor for the baseline (fixation cross inter-stimulus) were also included in the design matrix. For each subject, three first-level statistical contrast maps were computed: the direct contrasts between the three conditions and the baseline.

Second-level analysis was also performed to analyze the main effects of the group in each contrast by two-sample *t*-tests. Furthermore, one-sample *t*-tests were conducted to analyze the differences between conditions for each group separately. Whole-brain voxel-wise threshold of *p* < 0.001 was used and family-wise error (FWE) correction for multiple comparisons was applied at the cluster level with a threshold of *p* < 0.01.

#### Correlation analysis

Pearson’s correlations were conducted to examine the associations between brain activation in response to neutral stimuli linked to alcohol-related context in the BD group and various alcohol consumption variables (AUDIT score, age of BD onset, BD episodes per month, percentage of alcohol intoxication per drinking event, number of months with BD pattern, quantity of alcohol consumed—grams of alcohol consumed per week, and speed of drinking—grams of alcohol consumed per hour during BD episodes), as well as impulsivity traits measured by the BIS-11. These analyses aimed to assess potential interactions between alcohol use and impulsiveness with the activation levels in brain regions where significant differences were observed.

## Results

### Demographic data, drinking variables, and psychological traits comparisons

Significant differences between groups for all alcohol consumption variables (AUDIT scores, BD episodes per month, percentage of alcohol intoxication per drinking event, number of months with BD pattern, quantity of alcohol consumed—grams of alcohol consumed per week, and speed of drinking—grams of alcohol consumed per hour during BD episodes) were observed. These differences were anticipated given the nature and inclusion criteria of each group. In addition, BDs scored higher than controls for motor impulsivity on the BIS-11 subscale (*t* = 2.17, *p* < 0.05), and no differences were found between groups for BIS-11 main score, its remaining subscales, nor for any of the SCL-90-R subscales (see Table 2 and, for more details, refer to the Supplemental material). Lastly, a Chi-square test was conducted to assess the difference in sex distribution between the Control and the BD group. The test indicated no significant difference in gender distribution (χ²(1) = 0.562, *p* = 0.453). Thus, gender was not included as a covariate in the subsequent behavioral and neurofunctional analyses.

### Behavioral results

There were no significant behavioral group differences in RTs and correct recalls neither for both Alcohol and No-Alcohol conditions nor between conditions, for both the control and BD groups (see Table 3). Similarly, no differences were found between groups regarding correct inhibitions of the New stimuli.

**Table 3.** Behavioral data (mean (SD)).

	Correct response (%)			Reaction times (ms)	
Group	Alcohol	No-Alcohol	New	Alcohol	No-Alcohol
Binge drinkers	91.67 (12.59)	91.33 (13.79)	97.67 (0.07)	1218.19 (281.81)	1235.65 (311.03)
Controls	94.86 (4.98)	93.06 (5.84)	99.17 (0.02)	1214.42 (168.36)	1178.06 (189.44)

### Neuroimaging results

#### Group comparisons

**Figure 2.**
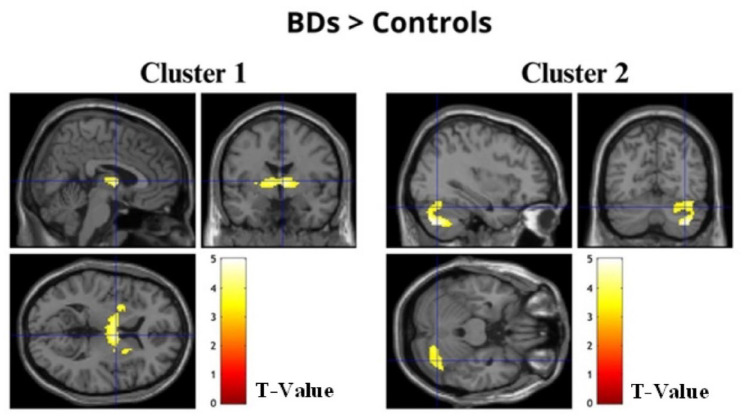
Images of the two clusters of regions displaying significantly increased activation in BDs when compared to controls for the alcohol condition.

Whole-brain analyses concerning the alcohol condition revealed increased BOLD activation in BDs during the recall of alcohol-related stimuli when compared with the control group. Specifically, two clusters of regions were identified: the first composed of the bilateral thalamus, globus pallidus, putamen, and right caudate nucleus (*t*(34) > 3.36, *p* (FWEc) < 0.01, 804 voxel extent threshold); and the second encompassed the right cerebellum and the fusiform gyrus (*t*(34) > 3.36, *p* (FWEc) < 0.01, 754 voxel extent threshold) (see Figure 2 and Table 4). No significative differences between groups were found for the No-Alcohol and the New conditions.

**Table 4.** Regions significantly activated at whole-brain analysis in the contrast BDs > Control Group for the Alcohol Condition showing significant (*p* (FWEc) < 0.01) BOLD signal changes, with local maxima more than 4.0 mm apart.

Region	*t*	Coordinates	*K*
Cluster 1			
Right thalamus	4.25	10, −6, 6	804
Left thalamus	4.25	−14, −12, 2	804
Left pallidum	4.20	−18, −6, 2	804
Right caudate nucleus	4.17	22, 16, 14	804
Left putamen	3.67	−28, 2, 4	804
Right pallidum	3.49	18, −4, 0	804
Right putamen	3.47	24, 6, 4	804
Cluster 2			
Right cerebellum Crus2	4.93	34, −66, −42	754
Right cerebellum Crus1	3.91	38, −72, −32	754
Right cerebellum_6	3.80	22, −72, −26	754
Right fusiform gyrus	3.87	40, −66, −20	754

#### Condition comparisons within groups

**Figure 3.**
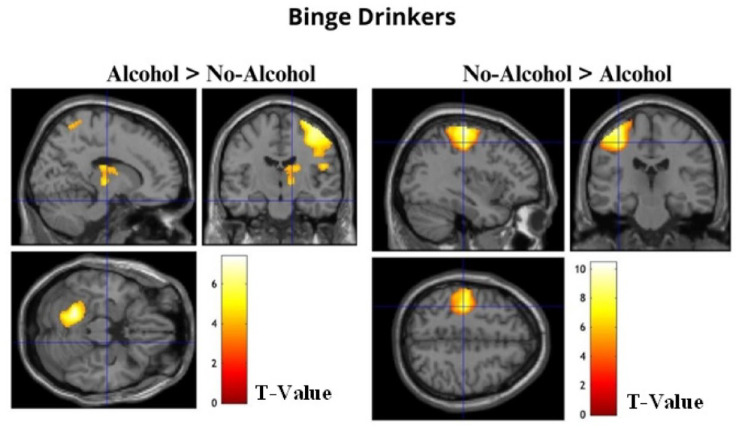
The images show significant activation maps of BDs for the contrasts Alcohol > No-Alcohol and No-Alcohol > Alcohol overlaid on an anatomical MRI image.

Whole-brain analyses concerning condition differences revealed that BDs exhibited increased BOLD activation when recalling Alcohol compared to No-Alcohol stimuli. Specifically, three clusters of regions were identified: the first composed of the right supramarginal, postcentral and precentral gyrus, rolandic operculum, and superior parietal lobule (*t*(19) > 3.58, *p* (FWEc) < 0.01, 3127 voxel extent threshold); the second encompassed the left fusiform gyrus and cerebellum (*t*(19) > 3.58, *p* (FWEc) < 0.01, 1124 voxel extent threshold); and the third included right thalamus and caudate nucleus (*t*(19) > 3.58, *p* (FWEc) < 0.01, 377 voxel extent threshold). In addition, BDs showed increased BOLD activation when recalling No-Alcohol compared to Alcohol stimuli in the left postcentral and precentral gyrus (*t*(19) > 3.58, *p* (FWEc) < 0.01, 2235 voxel extent threshold) (see Table 5 and Figure 3).

**Table 5.** Regions significantly activated at whole-brain analysis in the contrasts Alcohol > No-Alcohol and No-Alcohol > Alcohol for the BDs showing significant (*p* (FWEc) < 0.01) BOLD signal changes, with local maxima more than 4.0 mm apart.

Region	*t*	Coordinates	*K*
Alcohol > no-Alcohol			
Cluster 1			
Right precentral gyrus	6.88	38, −16, 56	3127
Right postcentral gyrus	6.73	40, −18, 48	3127
Right superior parietal lobule	5.84	26, −54, 64	3127
Right rolandic operculum	5.45	50, −18, 20	3127
Right supramarginal	4.03	58, −18, 20	3127
Cluster 2			
Left cerebelum_6	7.37	−22, −52, −22	1124
Left cerebelum_4_5	7.03	−18, −52, −20	1124
Left fusiform gyrus	4.72	−22, −52, −16	1124
Cluster 3			
Right thalamus	5.13	16, −18, 18	337
Right caudate nucleus	4.12	12, −6, 16	337
No-Alcohol > alcohol			
Left postcentral gyrus	10.40	−48, −20, 56	2235
Left precentral gyrus	9.92	−36, −20, 60	2235

**Figure 4.**
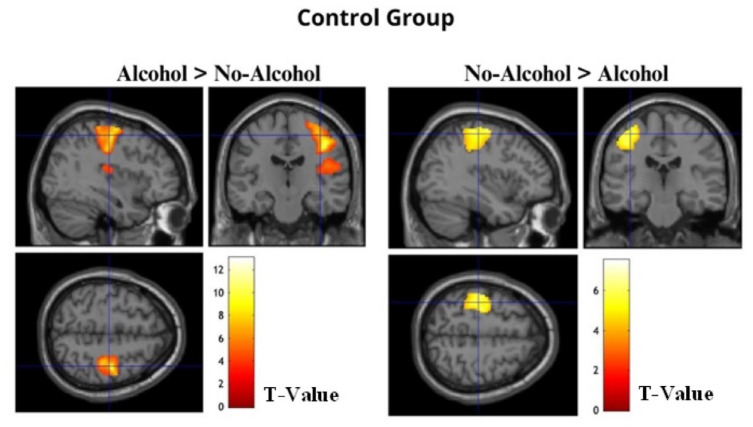
The images show significant activation maps of the Control Group for the contrasts Alcohol > No-Alcohol and No-Alcohol > Alcohol overlaid on an anatomical MRI image.

For the control group, the whole-brain analysis revealed increased BOLD activation when recalling Alcohol compared to No-Alcohol stimuli. Specifically, two clusters of regions were identified: the first composed of the right postcentral and precentral gyrus (*t*(15) > 3.79, *p* (FWEc) < 0.01, 1732 voxel extent threshold); and the second encompassed right supramarginal gyrus and rolandic operculum (*t*(15) > 3.79, *p* (FWEc) < 0.01, 400 voxel extent threshold). In addition, like BDs, the control group showed increased BOLD activation when recalling No-Alcohol compared to Alcohol stimuli in the left postcentral and precentral gyrus (*t*(15) > 3.79, *p* (FWEc) < 0.01, 1488 voxel extent threshold) (Table 6, Figure 4).

**Table 6.** Regions significantly activated at whole-brain analysis in the contrast Alcohol > No-Alcohol and No-Alcohol > Alcohol for the control group showing significant (*p* (FWEc) < 0.01) BOLD signal changes, with local maxima more than 4.0 mm apart.

Region	*t*	Coordinates	*K*
Alcohol > no-Alcohol			
Cluster 1			
Right precentral gyrus	8.52	34, −10, 60	1732
Right postcentral gyrus	13.01	46, −16, 46	1732
Cluster 2			
Right rolandic operculum	5.45	46, −18, 20	400
Right supramarginal	4.51	58, −18, 20	400
No-Alcohol > alcohol			
Left precentral gyrus	7.52	−32, −16, 66	1488
Left postcentral gyrus	6.35	−42, −22, 48	1488

### Correlations analysis

Pearson’s correlations between brain activations elicited by neutral stimuli linked to alcohol-related context in the BD group and alcohol consumption variables did not reveal any significant interactions (for more details, see Supplemental material). Regarding impulsivity, a significant correlation was found between the Self-Control subscale and activation in Cluster 3 (right Thalamus and Caudate Nucleus), when comparing Alcohol with No-Alcohol conditions for BDs (*r* = 0.522, *p* < 0.05). No further significant correlations were observed between brain activations in response to neutral stimuli associated with alcohol-related context and other impulsivity measures (for more details, see Supplemental material).

## Discussion

This study aimed to examine the impact of BD on brain mechanisms linked to alcohol-related associative memory in college students. Despite the absence of significant behavioral differences, results showed a brain hyperactivity pattern in young BDs during the recall of alcohol-related stimuli. Notably, BDs exhibited increased activity in two different clusters, including reward processing regions and areas related to memory and mental imagery, when compared with abstainers.

Specifically, BDs displayed greater activation in the bilateral thalamus, globus pallidus, and dorsal striatum (DS) (putamen and right caudate nucleus), in comparison to the control group, regions known to be affected by acute alcohol consumption (Patton et al., 2023), and involved in reward processing (Diekhof et al., 2008). Augmented activity in these areas in response to alcohol/drug cues has often been reported in individuals with heavy alcohol/drug use, including BDs (Cservenka & Brumback, 2017; Delgado, 2007; George et al., 2001; Vollstädt-Klein et al., 2010). In this sense, when exposed to alcohol-related cues, heavy drinkers have shown greater activity in visual, memory-related, and incentive salience regions, including the fusiform gyrus, thalamus, and striatum nuclei (Everitt, 2014; George et al., 2001; Vollstädt-Klein et al., 2010). Therefore, the increased activation of the thalamus, globus pallidus, and DS observed in BDs might be interpreted as the ability of neutral cues, when paired with alcohol contexts, to elicit enhanced brain activation in regions associated with craving and reward processing, even in the absence of the context itself.

Importantly, preclinical and clinical research has shown that during cue-reactivity tasks, casual consumers, or those who have recently started consuming alcohol or other drugs, display greater brain activation in the ventral striatum (Courtney et al., 2018; Porrino et al., 2007). Nonetheless, individuals who have transitioned from casual drug use to habitual/compulsive consumption show increased activity—and greater dopamine release—in the DS when exposed to substance-associated cues (Everitt and Robbins, 2013; Heinz et al., 2009; Vollstädt-Klein et al., 2010). In this sense, long-lasting associations between stimulus (drug) and response (craving and substance seeking) are mediated by the DS (Everitt and Robbins, 2005; Ito et al., 2002), and higher activation in the thalamic and striatal regions has been related to increased impulsivity as well as to reward sensitivity and processing (Fede et al., 2020; Moussawi et al., 2016). Therefore, it is not surprising that neural alterations in the DS are commonly observed in individuals with addictive behaviors (Koob & Volkow, 2016; Volkow et al., 2006). Furthermore, greater cue-induced activation in the DS has been shown to predict relapse in alcohol-dependent individuals (Bach et al., 2015; Grüsser et al., 2004), while relapse has been directly associated with higher alcohol craving levels (Sliedrecht et al., 2019; Stohs et al., 2019; Witteman et al., 2015). Accordingly, the results found in the current study, showing that BDs exhibit heightened brain activation primarily in the DS during the recall of alcohol contexts when compared with the control group, are in line with the assumption that BD might potentially be a transitional consumption pattern, bridging the gap between casual consumption and alcohol abuse (Almeida-Antunes et al., 2021; Enoch, 2006; Lannoy et al., 2019; Parsons, 1998).

Moreover, our results revealed that, in comparison with controls, BDs exhibited greater activation in the cerebellum as well as in the fusiform gyrus when recalling alcohol-related contexts. These two regions have been described as relevant in visual processing (Palejwala et al., 2020) and task-driven motor processing in response to a stimulus (Guell & Schmahmann, 2020). Specifically, the fusiform gyrus is involved in the processing of visual information and semantic memory (Danker and Anderson, 2010; Khader et al., 2005; Mion et al., 2010), particularly during memory recall and imagery of content-specific or scene-related stimuli (Johnson & Rugg, 2007; Spagna et al., 2021), reinforcing thus the idea that this region is prone to activation during associative memory processing, not only during encoding but also during the retrieval of previously associated stimuli (Dijkstra et al., 2019; Park et al., 2012). Likewise, the cerebellum, although traditionally described as being purely a motor control region, has also been described as a contributor to cognitive processing and emotional control (Baumann et al., 2015; Schmahmann and Caplan, 2006; Strata, 2015). Regarding drug consumption, it has often been considered as an intermediary between the motor reward and the motivational-cognitive control systems of the striatal-cortico-limbic circuitry (Moulton et al., 2014). Notably, the cerebellum appears to be a relevant node for drug-related cue association, playing significant roles in conditioning response, goal-directed behavior, and selective attention (Brumback et al., 2015; Gil-Miravet et al., 2019; Miquel et al., 2020; Moulton et al., 2014). These functions are integral to the constructs of craving and addictive behavior, as they influence how individuals respond to drug-related cues and the subsequent behavioral outcomes (Franken, 2003). In the same vein, when exposed to alcohol cues, brain activation in the cerebellum has been positively correlated with AUD through AUDIT scores (Al-Khalil et al., 2021). Therefore, the cerebellum’s engagement in these processes underscores its role as a significant hub for drug-related cue association. By integrating motor, cognitive, and emotional responses, the cerebellum mediates the processes that occur between observing environmental cues and the behavioral outcomes associated with addiction.

Consequently, the increased activity observed in these two regions—the fusiform gyrus and the cerebellum—in the BDs relative to the control group, might suggest that, in accordance with what has been described in the literature, BDs exhibit augmented neural reactivity when exposed to alcohol-related stimuli (Almeida-Antunes et al., 2022; Blanco-Ramos et al., 2022; Pérez-García et al., 2022; Petit et al., 2014). In detail, the heightened BDs’ BOLD activation in the fusiform gyrus appears to underline the recalling and mental imagery of scene-specific alcohol-related context, while the elevated activation in the cerebellum may be related to the selective attention and increased salience toward alcohol-related stimuli, often seen in BDs (Gordon, 2007; Johnson & Rugg, 2007). In parallel, this finding might indicate that the mental representation of the drinking context could elicit craving, aligning with previous research suggesting that increased activation in the fusiform gyrus and the cerebellum is directly associated with heightened cue-induced alcohol craving (Moreno-Rius and Miquel, 2017; Olbrich et al., 2006; Park et al., 2007; Schneider et al., 2001).

Comparison of the Alcohol and No-Alcohol conditions revealed increased activation in the primary sensorimotor cortex, contralateral to the hand used by participants to classify the stimuli, in both groups. Accordingly, when participants were presented with alcohol-related stimuli—identified by pressing a button with the left hand—increased activation was observed in the right precentral and postcentral gyri. Conversely, when they were presented with No-Alcohol stimuli—identified by pressing a button with the right hand—greater activation was seen in the left precentral and postcentral gyri reflecting thus the well-known contralateral motor effect (Allison et al., 2000). Moreover, BDs displayed increased BOLD activation in an additional cluster of regions when presented with alcohol-related stimuli compared to No-Alcohol stimuli. Namely, this heightened activation was observed in the thalamus, caudate nucleus, cerebellum, and fusiform gyrus—regions involved in the visual and reward networks (Guell & Schmahmann, 2020; Palejwala et al., 2020) that show increased cue reactivity to alcohol-related stimuli in heavy drinkers and BDs, as described above (Cservenka & Brumback, 2017; Schacht et al., 2013). This suggests that BD experiences intensified alcohol craving in response to neutral stimuli associated with drinking-related contexts, as indicated by increased brain activation in these areas, a response not observed in control participants.

Furthermore, a significant positive correlation was observed between the self-control subscale of the BIS-11 and brain activation in the thalamus and caudate nucleus when BDs were exposed to alcohol-related cues. Specifically, higher scores on this subscale, indicating lower self-control (Patton et al., 1995), were associated with increased activity in these brain regions. Given the thalamus’s role in processing sensory information (Shine, 2023) and the caudate nucleus’ involvement in reward-related processes (Delgado et al., 2004), these findings suggest that BDs with lower self-control may experience intensified cue-induced craving for alcohol, reflected by heightened brain activation in these areas. This relationship aligns with previous research concerning substance use disorders (Li et al., 2010), highlighting the potential link between diminished self-control and increased alcohol craving (Bernard et al., 2021).

At the behavioral level, both groups exhibited comparable performance in both the Alcohol and No-Alcohol conditions, displaying similar accuracy, that is, a number of correct recalls for both conditions. Since the primary objective of our study was to investigate the brain mechanisms involved in associative memory, we ensured a correct recall rate of at least 90% before conducting the task within the MRI scanner. Therefore, a ceiling effect may have influenced the results. In addition, previous studies suggest that through neural compensation, experimental groups (i.e., those with alcohol consumption) can exhibit similar behavioral performance as controls while showing higher brain activation (Almeida-Antunes et al., 2021; Bagga et al., 2014; Hatchard et al., 2017; Suárez-Suárez et al., 2020). Regarding task performance speed, while group comparisons revealed higher motor impulsivity—as measured by the BIS-11—for BDs compared to controls, there were no differences in reaction times between groups for either the Alcohol or No-Alcohol conditions. The tendency to act impulsively with little regard for negative consequences has been widely explored in BDs (Bø et al., 2016; Maurage et al., 2019), highlighting the pronounced reward-seeking and impulsive behaviors characteristic of this population (Lees et al., 2019). Literature has shown that high consumption patterns, such as BD, are associated with higher impulsivity (Balodis et al., 2009). Nevertheless, neuropsychological assessments as well as behavioral results derived from neuroimaging/EEG studies often lack the sensitivity to detect potential brain alterations caused by BD, even in the presence of neurofunctional differences between BDs and controls (Crego et al. 2009, 2012; Carbia et al., 2018).

The present study has some limitations that need to be addressed in future research. First, prior to, or during data collection, no measurement of subjective craving was assessed. Such a measure would have provided important information for understanding how brain activation in the clusters of interest could be related to craving, and how it may influence and/or be influenced by such associative memory processes. In addition, our sample comprised two polarized groups, including individuals at the two non-clinical polar ends of alcohol consumption, BDs and abstainers. Future investigation, including a group of light or social drinkers (non-harmful and casual consumption), might provide a more comprehensive understanding of the development of BD as a harmful consumption pattern and elucidate how and when craving begins to influence/be influenced by alcohol-related memories. Likewise, longitudinal research on the developmental trajectory of BD and its impact on the brain would be valuable for understanding potential causal effects. This research could further explore how these brain alterations evolve with continued alcohol use and whether they might revert after the cessation of BD episodes (Brumback et al., 2015; López-Caneda et al., 2014b). These insights would enhance our understanding of the *continuum* hypothesis between BD and AUD—the idea that BD and alcohol dependence may constitute successive stages of the same phenomenon (Enoch, 2006; Parsons, 1998). This hypothesis is supported by observed impairments in BDs similar to those seen in individuals with AUD (e.g., Crego et al., 2010; López-Caneda et al., 2017; Maurage et al., 2009; Petit et al., 2014; Sanhueza et al., 2011), suggesting that BD during adolescence might be a starting point for the development of alcohol dependence in adulthood (Bonomo et al., 2004; McCambridge et al., 2011; McCarty et al., 2004). Lastly, no data were collected regarding the menstrual cycle phase of female participants on the day of the fMRI scan. Recent literature indicates that menstrual cycle phases, particularly fluctuations in progesterone and estradiol levels, can influence brain dynamics (Avila-Varela et al., 2024), so future research should consider these hormonal variations to ensure more robust and reliable results.

In conclusion, despite the absence of significant behavioral differences between groups, neutral cues linked to alcohol-related contexts may trigger heightened brain activity in the BD population. Namely, our findings revealed augmented activation in two clusters comprising the bilateral thalamus and the striatum, as well as by the cerebellum and the fusiform gyrus. These regions have been associated with memory and mental imagery (Spagna et al., 2021; Mion et al., 2010), the processing of visual cues (Tsapkini, et al. 2011), and reward and craving modulation (Braus et al., 2001; Moulton et al., 2014; Paulus, 2007). Results are in line with existing literature indicating that prior consumption contexts significantly influence craving and relapse in alcohol-dependent individuals (Janak & Chaudhri, 2010). Moreover, our findings suggest that real-life contexts involving alcohol consumption (Kuerbis et al., 2020; Nees et al., 2012) may become linked or associated with otherwise neutral stimuli. Consequently, these newly associated stimuli could elicit brain responses similar to those triggered by direct exposure to alcohol-related contexts. This association might lead to an increase in craving and, subsequently, substance-seeking and taking behaviors (Papachristou et al., 2012; Weiss, 2005). Overall, while evidence remains limited, this study sheds light on the importance of alcohol-associated memories and their potential impact on craving and alcohol use in BDs. Future research on alcohol abuse and BD should consider the context of consumption and explore how those environmental cues might influence alcohol-seeking behavior and the maintenance or escalation of alcohol consumption.

## Supplemental Material

sj-docx-1-jop-10.1177_02698811241282624 – Supplemental material for Associative memory in alcohol-related contexts: An fMRI study with young binge drinkers Supplemental material, sj-docx-1-jop-10.1177_02698811241282624 for Associative memory in alcohol-related contexts: An fMRI study with young binge drinkers by Rui Pedro Serafim Rodrigues, Sónia Silva Sousa, Eduardo López-Caneda, Natália Almeida-Antunes, Alberto Jacobo González‑Villar, Adriana Sampaio and Alberto Crego in Journal of Psychopharmacology
