# Supplementary material for: Associative memory in alcohol-related contexts: An fMRI study with young binge drinkers
Source: J Psychopharmacol. 2024 Oct 7;38(11):972–85. doi: 10.1177/02698811241282624 (PMC11528936; doi:10.1177/02698811241282624)
Supplement: sj-docx-1-jop-10.1177_02698811241282624 – Supplemental material for Associative memory in alcohol-related contexts: An fMRI study with young binge drinkers [file sj-docx-1-jop-10.1177_02698811241282624.docx]

Supplementary Materials:

Table 1: Correlations between alcohol use measures and significative brain activation for each cluster.

|  | | AUDIT Score | BD events per month | Percentage of alcohol intoxication per drinking event | Quantity of alcohol consumed (gr/week) | Speed of drinking gr/h | Months with a BD pattern | Age onset of BD pattern |
| --- | --- | --- | --- | --- | --- | --- | --- | --- |
| Group Comparisons: BDs > Control Group for the Alcohol Condition | | | | | | | | |
| Cluster 1 – TH & DS | Pearson’s correlation | 0.104 | -0.225 | -0.310 | 0.055 | -0.310 | -0.055 | -0.075 |
|  | *p-*value | 0.671 | 0.340 | 0.184 | 0.819 | 0.184 | 0.818 | 0.669 |
| Cluster 2 – CB & FG | Pearson’s correlation | -0.205 | -0.084 | -0.074 | 0.183 | -0.157 | 0.002 | 0.037 |
|  | *p-*value | 0.400 | 0.726 | 0.757 | 0.441 | 0.509 | 0.993 | 0.835 |
| Condition Comparisons: Alcohol > No-Alcohol for the BDs | | | | | | | | |
| Cluster 3 – TH & CN | Pearson’s correlation | 0.154 | 0.149 | 0.184 | -0.110 | -0.307 | -0.262 | -0.019 |
|  | *p-*value | 0.530 | 0.531 | 0.437 | 0.644 | 0.188 | 0.265 | 0.937 |

Note: TH – Thalamus; DS – Dorsal Striatum; CB – Cerebellum; FG – Fusiform Gyrus; CN – Caudate Nucleus.

Table 2: Correlations between significative brain activation for each cluster with BIS11 score and each subscale.

|  | | Total Score | Attention | Cognitive Instability | Motor | Perseverance | Self-Control | Cognitive Complexity |
| --- | --- | --- | --- | --- | --- | --- | --- | --- |
| Group Comparisons: BDs > Control Group for the Alcohol Condition | | | | | | | | |
| Cluster 1 – TH & DS | Pearson’s correlation | -0.101 | -0.275 | -0.017 | -0.083 | -0.074 | 0.124 | 0.026 |
|  | *p-*value | 0.671 | 0.241 | 0.944 | 0.727 | 0.755 | 0.604 | 0.914 |
| Cluster 2 – CB & FG | Pearson’s correlation | -0.167 | -0.132 | -0.017 | -0.212 | -0.015 | 0.059 | -0.101 |
|  | *p-*value | 0.482 | 0.578 | 0.944 | 0.370 | 0.951 | 0.806 | 0.672 |
| Condition Comparisons: Alcohol > No-Alcohol the BDs | | | | | | | | |
| Cluster 3 – TH & CN | Pearson’s correlation | -0.141 | -0.250 | -0.213 | -0.394 | 0.181 | 0.522* | -0.179 |
|  | *p-*value | 0.553 | 0.289 | 0.367 | 0.086 | 0.446 | 0.018 | 0.449 |

Note: TH – Thalamus; DS – Dorsal Striatum; CB – Cerebellum; FG – Fusiform Gyrus; CN – Caudate Nucleus; * p < 0.05.

Table 3: Group Comparisons between Controls and Binge Drinkers for each SCL-90 Index for both sexes.

| Measure | Group | Whole Sample  Mean (SD) | P-value | Females  Mean (SD) | P-value | Males  Mean (SD) | P-value |
| --- | --- | --- | --- | --- | --- | --- | --- |
| Somatization | Control | 0.518 (0.463) | 0.338 | 0.675 (0.528) | 0.268 | 0.258 (0.162) | 0.265 |
|  | BD | 0.673 (0.480) |  | 0.920 (0.434) |  | 0.425 (0.402) |  |
| Obsessive compulsive | Control | 1.106 (0.491) | 0.183 | 1.168 (0.440) | 0.122 | 1.002 (0.596) | 0.577 |
|  | BD | 1.327 (0.482) |  | 1.485 (0.433) |  | 1.170 (0.498) |  |
| Interpersonal sensitivity | Control | 0.959 (0.564) | 0.793 | 1.212 (0.450) | 0.326 | 0.528 (0.499) | 0.259 |
|  | BD | 0.917 (0.402) |  | 1.012 (0.437) |  | 0.822 (0.360) |  |
| Depression | Control | 1.006 (0.640) | 0.825 | 1.164 (0.659) | 0.611 | 0.743 (0.562) | 0.634 |
|  | BD | 0.960 (0.597) |  | 1.025 (0.540) |  | 0.896 (0.672) |  |
| Anxiety | Control | 0.781 (0.611) | 0.716 | 0.893 (0.701) | 0.420 | 0.595 (0.411) | 0.764 |
|  | BD | 0.580 (0.577) |  | 0.631 (0.720) |  | 0.530 (0.422) |  |
| Hostility | Control | 0.588 (0.551) | 0.318 | 0.841 (0.554) | 0.418 | 0.167 (0.134) | 0.162 |
|  | BD | 0.480 (0.340) |  | 0.669 (0.352) |  | 0.291 (0.202) |  |
| Phobic anxiety | Control | 0.323 (0.304) | 0.953 | 0.386 (0.297) | 0.918 | 0.219 (0.312) | 0.336 |
|  | BD | 0.329 (0.233) |  | 0.373 (0.230) |  | 0.284 (0.241) |  |
| Paranoid Ideation | Control | 0.879 (0.524) | 0.437 | 0.864 (0.452) | 0.774 | 0.904 (0.674) | 0.194 |
|  | BD | 0.773 (0.369) |  | 0.916 (0.343) |  | 0.630 (0.353) |  |
| Psychoticism | Control | 0.498 (0.268) | 0.482 | 0.574 (0.226) | 0.332 | 0.373 (0.306) | 0.105 |
|  | BD | 0.516 (0.321) |  | 0.448 (0.331) |  | 0.585 (0.313) |  |
| Global Severity Index | Control | 0.618 (0.380) | 0.834 | 0.679 (0.414) | 0.368 | 0.517 (0.323) | 0.397 |
|  | BD | 0.647 (0.415) |  | 0.853 (0.426) |  | 0.442 (0.298) |  |
| Positive Symptom Total | Control | 38.492 (17.073) | 0.825 | 41.204 (15.742) | 0.246 | 33.972 (19.729) | 0.173 |
|  | BD | 38.293 (15.410) |  | 33.199 (14.070) |  | 43.387 (15.675) |  |
| Positive Symptom Distress Index | Control | 1.570 (0.393) | 0.474 | 1.613 (0.403) | 0.523 | 1.499 (0.402) | 0.449 |
|  | BD | 1.485 (0.333) |  | 1.497 (0.389) |  | 1.474 (0.287) |  |
